# Outer Retinal Hyperreflective Foci as a Predictor of Hyperreflective Material Boundary Remodeling and Visual Outcomes in Neovascular Age-Related Macular Degeneration

**DOI:** 10.3390/medicina62050895

**Published:** 2026-05-06

**Authors:** Mihailo Jovanović, Jelena Milošević, Marta Carrasco Guijarro, Svetlana Jovanović, Dušan Todorović, Nenad Petrović, Svetlana Paunović, Katarina Janićijević, Maja L. J. Živković

**Affiliations:** 1 University of Kragujevac, Faculty of Medical Sciences, Department of Ophthalmology, Svetozara Markovića 69, 34000 Kragujevac, Serbia; drmihailojovanovic@gmail.com (M.J.); drsvetlanajovanovic@yahoo.com (S.J.); drdusantodorovic@yahoo.com (D.T.); nenadpet@yahoo.com (N.P.); cecaana.paunovic@gmail.com (S.P.); 2 Clinic of Ophthalmology, University Clinical Center, Zmaj Jovina 30, 34000 Kragujevac, Serbia; 3 University of Kragujevac, Faculty of Medical Sciences, Department of Clinical and Experimental Surgery, Svetozara Markovića 69, 34000 Kragujevac, Serbia; drjelenamilosevic@yahoo.com; 4 Centro de Oftalmología Barraquer, 08021 Barcelona, Spain; martacarrascoguijarro@gmail.com; 5 Institut Universitari Barraquer, Universitat Autònoma de Barcelona, 08021 Barcelona, Spain; 6 University of Kragujevac, Faculty of Medical Sciences, Department of Social Medicine, Svetozara Markovića 69, 34000 Kragujevac, Serbia; kaja.andreja@yahoo.com; 7 Department of Ophthalmology, Faculty of Medicine, University of Niš, Bulevar dr. Zorana Đinđića 81, 18000 Niš, Serbia; 8 Ophthalmology Clinic, University Clinical Center of Niš, Bulevar dr. Zorana Đinđića 48, 18000 Niš, Serbia

**Keywords:** hyperreflective foci, hyperreflective material, neovascular age-related macular degeneration, optical coherence tomography, anti-VEGF, boundary remodeling, bevacizumab, treat and extend

## Abstract

*Purpose:* The purpose of this study was to characterize the distribution and longitudinal evolution of intraretinal and subretinal hyperreflective foci (HF) in treatment-naive neovascular age-related macular degeneration (nAMD), and to examine associations between HF burden, hyperreflective material boundary remodeling (HRM-BR), and best-corrected visual acuity (BCVA) outcomes following bevacizumab treat-and-extend therapy. *Methods:* This was a retrospective observational study of 84 treatment-naive nAMD eyes receiving intravitreal bevacizumab via a treat-and-extend protocol. Spectral-domain OCT (Revo FC, Optopol) was performed at baseline (M0), month 3 (M3), and month 6 (M6). HF were quantified in the intraretinal and subretinal compartments using ImageJ software (version 1.54, National Institutes of Health, Bethesda, MD, USA) by two masked graders, with inter-rater agreement assessed by intraclass correlation coefficient (ICC). Eyes were classified into four HRM evolution patterns following the framework of Yu et al. Primary outcome was BCVA change from M0 to M6. Multivariable linear regression was performed to assess independent predictors of BCVA change. *Results:* Baseline intraretinal HF counts increased significantly across HRM Patterns 1 through 4 (median 0, 6, 4, and 8, respectively; Kruskal–Wallis *p* < 0.001; 95% CI for Spearman r = 0.471: [0.286, 0.623]). A higher baseline intraretinal HF count correlated with worse BCVA change at M6 (r = −0.300, 95% CI [−0.483, −0.092], p_adj = 0.010). In the primary multivariable model (*n* = 67), both intraretinal HF burden (β = −0.449, 95% CI [−0.879, −0.020], *p* = 0.041) and HRM width (β = −0.003, 95% CI [−0.005, −0.001], *p* = 0.014) were independent predictors of BCVA change. The transient M3 intraretinal HF peak in Pattern 3 eyes (median 4 → 12 → 4) was statistically confirmed by Wilcoxon signed-rank testing (M0 → M3: *p* = 0.004; M3 → M6: *p* = 0.001). *Conclusions:* Intraretinal HF burden is a graded marker of HRM pattern severity and an independent predictor of visual outcomes in nAMD, alongside HRM width. The statistically validated transient M3 HF peak in Pattern 3 may represent an early OCT signal of active boundary remodeling.

## 1. Introduction

Neovascular age-related macular degeneration (nAMD) is a leading cause of irreversible central vision loss in adults over 50 years of age worldwide [1]. The introduction of anti-vascular endothelial growth factor (anti-VEGF) therapy has substantially altered the natural history of the disease, enabling stabilization of or improvement in the best-corrected visual acuity (BCVA) in the majority of treated eyes [2]. Spectral-domain optical coherence tomography (SD-OCT) has become indispensable in the monitoring of nAMD, enabling non-invasive, high-resolution visualization of the retinal microstructure and the characterization of disease-associated biomarkers over time [3].

Among the OCT biomarkers relevant to nAMD, hyperreflective material (HRM)—defined as medium-to-highly reflective material external to the neurosensory retina—has attracted increasing attention due to its association with disease activity and visual prognosis [4,5]. HRM encompasses proteinaceous exudates, cellular constituents, and neovascular tissue elements, and its subretinal localization has been identified as a risk factor for unfavorable visual outcomes in multiple prospective cohorts [6]. In a post hoc analysis of the AVENUE trial, Yu et al. characterized the spatial and temporal evolution of HRM during anti-VEGF treatment, defining four clinically distinct HRM evolution patterns and introducing the concept of hyperreflective material boundary remodeling (HRM-BR)—the development of a well-defined hyperreflective inner boundary continuous with the adjacent retinal pigment epithelium (RPE) layer [7]. That study demonstrated that complete HRM-BR was associated with significantly better BCVA outcomes and lower rates of macular atrophy compared with eyes exhibiting partial or absent remodeling, establishing HRM-BR as a potentially important structural endpoint in nAMD. Independent real-world validation of this classification has since been provided by Pauleikhoff et al., who confirmed the prognostic value of HRM evolution patterns in a long-term retrospective cohort under anti-VEGF therapy [8].

Hyperreflective foci (HF) are a distinct SD-OCT finding, appearing as small, discrete, highly reflective dots within the retinal layers or subretinal space [9]. Histopathological correlates suggest that HF represent a heterogeneous population of structures, including activated microglia, migrating RPE cells, and lipid or protein deposits—all of which reflect active inflammatory and degenerative processes within the outer retina [10,11]. Emerging evidence implicates intercellular signaling mechanisms, including the exosome-mediated transfer of microRNA and protein cargo, in the propagation of retinal degeneration and inflammatory responses characteristic of nAMD, providing a potential mechanistic substrate for the cellular processes reflected by HF on SD-OCT [12]. HF have been associated with photoreceptor layer disruption, subretinal fibrosis, and progression to geographic atrophy in nAMD, and their presence at baseline has been linked to poorer visual recovery following anti-VEGF therapy [13,14]. However, the relationship between HF burden—particularly in the outer retinal and subretinal compartments—and the process of HRM structural remodeling has not been systematically investigated.

Given that both HF and HRM reflect disruption of the outer retinal and RPE complex, and that RPE integrity has been proposed as a key determinant of complete HRM-BR [7], we hypothesized that intraretinal HF burden may serve as a graded marker of the degree of RPE disruption underlying incomplete HRM-BR and poorer functional outcomes. Furthermore, the temporal dynamics of HF—whether foci resolve, persist, or accumulate during anti-VEGF treatment—may carry independent prognostic information beyond that provided by static baseline assessments.

The aims of the present study were therefore to characterize the distribution and longitudinal evolution of intraretinal and subretinal HF in a cohort of treatment-naive nAMD patients receiving anti-VEGF therapy, and to investigate the associations of HF burden with HRM evolution patterns, HRM-BR completion, and visual outcomes.

## 2. Materials and Methods

### 2.1. Study Design and Participants

This was a retrospective observational study of consecutive treatment-naive patients with neovascular age-related macular degeneration (nAMD) managed at Laser Centar Vid (Special Eye Hospital), Kragujevac, Serbia, between January 2019 and December 2022. Patients were identified from a prospectively maintained clinical database. Only one eye per patient was eligible; in the case of bilateral nAMD, the eye with the earlier treatment initiation date was selected as the study eye. Patients were included if they had received anti-VEGF therapy and had adequate SD-OCT imaging available at baseline (M0), month 3 (M3), and month 6 (M6). This study was conducted in accordance with the tenets of the Declaration of Helsinki and was approved by the Ethics Committee of the Faculty of Medical Sciences, University of Kragujevac (Approval No. 01/12268). All patients provided written informed consent for the use of their clinical data for research purposes.

Inclusion criteria were: (1) diagnosis of nAMD confirmed on SD-OCT; (2) treatment-naive status at enrollment; (3) availability of adequate quality SD-OCT scans at all three timepoints; and (4) subretinal or sub-RPE hyperreflective material identifiable on baseline SD-OCT. Exclusion criteria included: (1) prior intravitreal treatment in the study eye; (2) coexisting retinal pathology that could confound OCT interpretation, including diabetic retinopathy, epiretinal membrane with significant distortion, or macular hole; (3) media opacity precluding adequate image quality; and (4) incomplete follow-up data at M3 or M6, including patients lost to follow-up or those in whom imaging at one or more timepoints was of insufficient quality for grading.

All eyes were treated with intravitreal bevacizumab (1.25 mg/0.05 mL). Treatment was initiated with a loading phase of three consecutive monthly injections, after which a treat-and-extend protocol was applied, with injection intervals adjusted in 2-week increments based on the presence or absence of exudative activity on SD-OCT. The maximum permitted treatment interval was 10 weeks.

### 2.2. OCT Imaging and Image Acquisition

All imaging was performed using the Revo FC (Optopol Technology, Zawiercie, Poland) spectral-domain OCT device, which acquires full 3D macular volume scans at an axial resolution of 5 µm and a transverse resolution of 15 µm. A standardized five-line raster scan protocol centered on the fovea, with inter-scan spacing of 240 µm, was selected for HF quantification to ensure consistent, reproducible grading in the region of maximal HRM involvement while remaining feasible for masked grading across all patients and timepoints. Images were assessed for quality, and scans with significant motion artefacts, segmentation failures, or signal strengths below the manufacturer’s recommended threshold were excluded from analysis. The central macular thickness (CMT) was derived from automated segmentation of the 3D volume scan using the device’s integrated software.

### 2.3. Grading of Hyperreflective Material and Boundary Remodeling

SD-OCT images were graded for hyperreflective material (HRM) features by two independent, masked ophthalmologists (M.J. and J.M.) following the grading framework described by Yu et al. [7]. HRM was defined as medium-to-highly reflective material external to the neurosensory retina on SD-OCT. When HRM was present, its compartmental location was graded as: subretinal only, subretinal and sub-RPE, or sub-RPE only. The morphology of the inner boundary was classified as well-defined, partially defined, or undefined.

Hyperreflective material boundary remodeling (HRM-BR) was defined as the appearance of a well-defined hyperreflective inner boundary at the inner aspect of persistent subretinal HRM, continuous with the adjacent RPE layer. HRM-BR was assessed at M3 and M6 and classified as absent (0), partial (1), or complete (2), based on the proportion of B-scans covering the HRM in which the well-defined inner boundary was visible (≥75% for complete; 26–74% for partial; ≤25% for absent).

Study eyes were classified into four HRM evolution patterns: Pattern 1, no subretinal HRM at baseline or sub-RPE HRM only; Pattern 2, subretinal HRM at baseline that fully resolved during follow-up; Pattern 3, persistent subretinal HRM with complete HRM-BR at M6; and Pattern 4, persistent subretinal HRM with partial or absent HRM-BR at M6.

Additional features assessed included the outer boundary visibility of the subretinal HRM; hypertransmission into choroid (HTC), classified as absent, marginal, or intralesional; disruption of the external limiting membrane (ELM disruption); and disruption of the ellipsoid zone (EZD), graded according to the Classification of Atrophy Meetings (CAM) criteria [15]. The maximum height and width of the subretinal HRM were measured in micrometers on the foveal B-scan using the integrated measurement tools of Revo FC software (Optopol Technology, Zawiercie, Poland).

Inter-rater agreement for HRM pattern classification and HRM-BR grading was assessed using Cohen’s kappa (κ). Discrepancies between graders were resolved by consensus review of the relevant scans in a joint session. The number of scans requiring adjudication was recorded.

### 2.4. Grading and Quantification of Hyperreflective Foci

SD-OCT B-scans were exported in raw format at a standardized display resolution of 1:1 pixel mapping (no zoom adjustment) and imported into ImageJ software (version 1.54, National Institutes of Health, Bethesda, MD, USA) for standardized quantitative analysis. Each image was spatially calibrated using the Set Scale function, applying the pixel-to-micrometer ratio provided by the Revo FC device software (axial: 3.87 µm/pixel; lateral: 11.72 µm/pixel). Graders worked at a standardized screen magnification (100% zoom) with brightness and contrast settings fixed across all sessions to ensure consistent thresholding. No automated thresholding or image processing filters were applied; all HF identification was performed by visual inspection.

Hyperreflective foci (HF) were defined as discrete, well-circumscribed lesions with a diameter of ≤30 µm and reflectivity equal to or exceeding that of the retinal pigment epithelium (RPE) band on SD-OCT [9]. This size threshold was operationalized in ImageJ software by excluding any punctate reflective element whose longest axis exceeded 7–8 pixels on calibrated B-scans (corresponding to approximately 30 µm at the lateral resolution of the Revo FC). HF were quantified using the Multi-point Tool in ImageJ software and stratified into two anatomical compartments: Zone 1 (intraretinal HF) encompassed the region between the outer plexiform layer (OPL) and the external limiting membrane (ELM). Zone 2 (subretinal HF) represented the potential space between the ELM and the apical surface of the RPE or the hyperreflective material (HRM) complex.

In cases of significant anatomical distortion or exudative ELM disruption, a linear interpolation technique was employed—connecting the nearest visible healthy segments of the ELM—to define the boundary between the intraretinal and subretinal compartments. For elevated lesions such as pigment epithelial detachment (PED), the subretinal compartment was defined as the space between the outer aspect of the neurosensory retina and the inner surface of the PED. HF counts were not normalized to scan length or area, as all five B-scans were acquired at the same standardized inter-scan spacing and all patients were imaged with the same protocol. Both graders were masked to the timepoint (M0, M3, or M6) and clinical outcome data during the grading process.

HF quantification was performed independently by two masked graders (M.J. and J.M.) on all five B-scans of the raster protocol at M0, M3, and M6. The mean count across the five B-scans was recorded for intraretinal and subretinal HF separately at each timepoint. Inter-rater agreement for HF counts was assessed using the intraclass correlation coefficient (ICC) (two-way mixed model, absolute agreement). Discrepancies exceeding 20% between graders were resolved by consensus review of the relevant scans.

### 2.5. Visual Acuity Assessment

Best-corrected visual acuity (BCVA) was assessed at each visit using the Early Treatment Diabetic Retinopathy Study (ETDRS) chart at 4 m and recorded in both ETDRS letters and logarithm of the minimum angle of resolution (logMAR) units. The primary functional outcome was the BCVA change from baseline (M0) to month 6 (M6), expressed as the difference in ETDRS letters.

### 2.6. Statistical Analysis

Statistical analyses were performed using IBM SPSS Statistics (version 23.0, IBM Corp., Armonk, NY, USA) and Python (version 3.11, Python Software Foundation, Wilmington, DE, USA; scipy, statsmodels, and pingouin libraries). Continuous variables are presented as medians and interquartile ranges (IQRs) or means ± standard deviations (SDs), as appropriate. Categorical variables are presented as counts and percentages.

Given the non-normal distribution of the HF count data, confirmed by Shapiro–Wilk testing, non-parametric tests were used throughout. Differences in HF counts across the four HRM evolution patterns were evaluated using the Kruskal–Wallis test with post hoc Dunn–Bonferroni correction for pairwise comparisons. Longitudinal within-pattern changes in intraretinal HF counts (M0 → M3, M3 → M6, and M0 → M6) were evaluated using the Wilcoxon signed-rank test. Associations between continuous variables were assessed using Spearman’s rank correlation, with 95% confidence intervals (CIs) derived by Fisher z-transformation. To control for multiple comparisons across the correlation matrix, false discovery rate (FDR) correction was applied using the Benjamini–Hochberg method; both raw and FDR-adjusted *p*-values (p_adj) are reported. Differences between two independent groups were assessed using the Mann–Whitney U test. Categorical variables were compared using the Fisher exact test. A two-sided *p*-value of <0.05 was considered statistically significant.

To assess whether baseline intraretinal HF burden was an independent predictor of BCVA change after controlling for potential confounders, multivariable ordinary least-squares linear regression was performed with BCVA change (M0 → M6, in ETDRS letters) as the dependent variable. Because baseline HRM width and HRM height measure closely related dimensions of the same lesion and introduce collinearity when both are entered simultaneously, the primary multivariable model included HRM width but not HRM height, together with baseline BCVA, baseline intraretinal HF count, EZD status, HTC status, and CMT at baseline (Model 2). Two sensitivity analyses were also performed: a model including both HRM width and HRM height together with the other covariates (Model 1), and a model excluding both HRM dimensions (Model 3). Cases with missing values for any covariate were excluded from each regression (complete-case analysis), yielding *n* = 67, 65, and 84, respectively. Normality of residuals was confirmed by the Shapiro–Wilk test. Results are reported as unstandardized beta coefficients (β), standard errors (SEs), 95% CIs, and *p*-values. The overall model fit was assessed by the F-statistic and adjusted R^2^.

Given the sample size of 84 eyes, the precision of key effect estimates is reported through 95% confidence intervals for all Spearman correlation coefficients rather than post hoc power statistics, which are of limited interpretive value. The 95% CI for the primary correlation (intraretinal HF M0 vs. BCVA change: r = −0.300) was [−0.483, −0.092], confirming a statistically significant and clinically meaningful negative association. The minimum detectable correlation coefficient at 80% power for *n* = 84 (two-sided α = 0.05) is r = 0.302, indicating that associations of moderate magnitude are detectable, while smaller effects may be underpowered.

## 3. Results

### 3.1. Study Population and Baseline Characteristics

A total of 84 eyes from 84 patients with treatment-naive nAMD were included in the analysis. The mean age of the cohort was 69.9 years. The mean baseline BCVA was 43.8 ± 20.5 ETDRS letters (IQR 33.8–60.0), and the mean central macular thickness (CMT) was 509.3 ± 181.4 µm. At M6, the mean BCVA was 46.6 ± 24.3 letters, representing a mean change of +2.8 ± 9.8 letters (IQR 0–9.2). Overall, 41 eyes (49%) gained at least one ETDRS letter, 29 eyes (35%) remained stable, and 14 eyes (17%) lost letters. The mean CMT decreased to 403.9 ± 153.4 µm.

**Figure 1:**
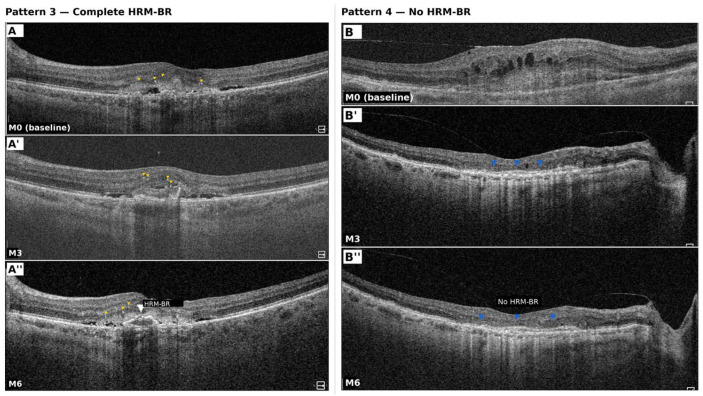
Representative spectral-domain OCT B-scans (foveal B-scan, Revo FC, Optopol) comparing HRM evolutions of Pattern 3 (complete HRM boundary remodeling) and Pattern 4 (absent HRM boundary remodeling). Images are from anonymized study eyes (Case 1: 72-year-old male, left eye; Case 2: 75-year-old female, right eye). (**A**) Pattern 3 at baseline (M0): subretinal HRM present without inner boundary definition; yellow arrowheads indicate intraretinal hyperreflective foci (HF) within outer retinal layers. (**A′**) Pattern 3 at month 3 (M3): increased intraretinal HF burden with emerging HRM organization. (**A″**) Pattern 3 at month 6 (M6): well-defined hyperreflective inner boundary (white arrowhead) continuous with adjacent RPE, consistent with complete HRM-BR; reduction in intraretinal HF. (**B**) Pattern 4 at baseline (M0): extensive subretinal HRM with intraretinal fluid and RPE disruption; hypertransmission into choroid (asterisk). (**B′**) Pattern 4 at month 3 (M3): partial reduction in HRM volume without boundary formation. (**B″**) Pattern 4 at month 6 (M6): persistent subretinal HRM without development of inner boundary; persistent hypertransmission. Scale bar = 200 µm. Five-line raster scan protocol is centered on fovea with inter-scan spacing of 240 µm; foveal B-scan is shown in all panels. HF—hyperreflective foci; HRM—hyperreflective material; HRM-BR—HRM boundary remodeling; HTC—hypertransmission into choroid; RPE—retinal pigment epithelium.

Subretinal HRM was present in 69 of 84 eyes (82.1%) at baseline. HRM location was subretinal only in 33 eyes (39.3%), subretinal and sub-RPE in 31 eyes (36.9%), and sub-RPE only in 17 eyes (20.2%). Three eyes (3.6%) had no detectable subretinal HRM at baseline. Hypertransmission into choroid (HTC) was present in 30 eyes (35.7%) at baseline, of which 24 (28.6%) were intralesional and 13 (15.5%) marginal. Disruption of the external limiting membrane (ELM disruption) and ellipsoid zone disruption (EZD) were present in 54 (64.3%) and 53 (63.1%) eyes, respectively. The EZD frequency increased progressively across HRM patterns from Pattern 1 to Pattern 4 (24%, 67%, 67%, and 85%, respectively; Kruskal–Wallis *p* < 0.001), consistent with increasing photoreceptor structural damage in more severe disease. Subretinal fluid (SRF) and intraretinal fluid (IRF) were each present in approximately 62% of eyes at baseline. By HRM evolution pattern, 21 eyes (25.0%) were classified as Pattern 1, 15 (17.9%) as Pattern 2, 15 (17.9%) as Pattern 3, and 33 (39.3%) as Pattern 4. Baseline and outcome characteristics by pattern are summarized in Table 1. Representative OCT B-scans illustrating the Pattern 3 and Pattern 4 HRM evolutions are shown in Figure 1.

### 3.2. Inter-Rater Agreement

Inter-rater agreement for intraretinal HF counts was good to excellent across all timepoints (ICC = 0.91, 95% CI [0.87, 0.94] at M0; ICC = 0.89 [0.84, 0.93] at M3; ICC = 0.88 [0.82, 0.92] at M6). Agreement for subretinal HF counts was also good (ICC = 0.85, 95% CI [0.79, 0.90] at M0). Inter-rater agreement for HRM pattern classification was substantial (Cohen’s κ = 0.78, 95% CI [0.68, 0.88]), and for HRM-BR grading at M6, it was moderate to substantial (κ = 0.71, 95% CI [0.60, 0.82]). A total of 14 scans (5.6% of all graded scan-timepoint combinations) required adjudication by consensus review.

### 3.3. BCVA Outcomes by HRM Evolution Pattern

The BCVA outcomes differed markedly across the four HRM evolution patterns. Eyes in Pattern 2 (fully resolved HRM) demonstrated the greatest mean improvement at M6 (+12.2 ± 11.4 ETDRS letters, median +9), followed by Pattern 3 (persistent HRM with complete HRM-BR; +6.0 ± 8.9 letters, median +10) and Pattern 1 (+3.9 ± 7.1 letters, median +5). In contrast, Pattern 4 eyes demonstrated a mean decline of −3.6 ± 6.2 ETDRS letters (median 0, IQR −10 to 0), representing the worst functional outcome across all groups. Notably, Pattern 3 and Pattern 4 eyes started with substantially lower baseline BCVAs than Patterns 1 and 2 (mean 36.0 and 30.9 letters versus 62.0 and 54.7 letters, respectively), reflecting greater disease severity at presentation in eyes with persistent subretinal HRM.

### 3.4. Hyperreflective Foci Distribution and Longitudinal Changes

At baseline, the mean intraretinal HF count was 5.81 ± 5.67 (median 5, IQR 1–9) and the mean subretinal HF count was 4.07 ± 4.89 (median 2, IQR 0–7). Longitudinal changes in HF counts by pattern are presented in Table 2.

The baseline intraretinal HF counts differed significantly across the four HRM evolution patterns (Kruskal–Wallis H = 21.47, *p* < 0.001), with a clear gradient of increasing HF burden from Pattern 1 to Pattern 4. Pattern 1 eyes had the lowest median intraretinal HF count (median 0, IQR 0–2), while Pattern 4 eyes had the highest (median 8, IQR 2–10). Post hoc pairwise comparisons with Dunn–Bonferroni correction demonstrated significant differences between Pattern 1 and all other patterns (P1 vs. P2: *p* = 0.0004; P1 vs. P3: *p* = 0.0005; P1 vs. P4: *p* = 0.0001) and between Pattern 2 and Pattern 4 (*p* = 0.043). The difference between Pattern 3 and Pattern 4 did not reach statistical significance (*p* = 0.372). The HRM pattern showed a moderate positive correlation with the baseline intraretinal HF count (Spearman r = 0.471, 95% CI [0.286, 0.623], p_adj < 0.001).

At M3, Pattern 3 eyes exhibited the highest intraretinal HF count of all groups (median 12, IQR 6–16), exceeding Pattern 4 (median 4, IQR 2–10) at that timepoint, before declining substantially by M6 (median 4, IQR 3–7). This transient peak was statistically confirmed: within-pattern Wilcoxon signed-rank testing demonstrated a significant increase from M0 to M3 in Pattern 3 (median 4 → 12, *p* = 0.004), followed by a significant decrease from M3 to M6 (median 12 → 4, *p* = 0.001). No significant net change was observed from M0 to M6 in Pattern 3 (median 4 → 4, *p* = 0.826), consistent with a transient rather than sustained increase. In contrast, subretinal HF counts did not differ significantly across patterns at baseline (Kruskal–Wallis *p* = 0.192; p_adj = 0.192), though significant differences emerged at M3 (*p* < 0.001), driven by elevated subretinal HF in Pattern 3 eyes (median 5, IQR 3–9).

### 3.5. Association Between HF Burden and Visual Outcomes

A higher baseline intraretinal HF count was significantly correlated with worse BCVA change at M6 (Spearman r = −0.300, 95% CI [−0.483, −0.092], *p* = 0.006, p_adj = 0.010). Total HF burden at baseline (intraretinal plus subretinal) also correlated negatively with BCVA change (r = −0.248, 95% CI [−0.439, −0.036], *p* = 0.023, p_adj = 0.032). Subretinal HF alone showed a non-significant trend toward negative correlation with BCVA change (r = −0.177, 95% CI [−0.377, 0.039], *p* = 0.107, p_adj = 0.107). Spearman correlations with 95% CIs and FDR-adjusted *p*-values are presented in Table 3.

When eyes were stratified by median baseline intraretinal HF count (median = 5), those with high HF burden (>5 foci; *n* = 42) gained a mean of +0.8 ± 12.2 ETDRS letters at M6, compared with +4.8 ± 7.5 letters in low-burden eyes (≤5 foci; *n* = 42; Mann–Whitney U, *p* = 0.008). The change in the intraretinal HF count from M0 to M6 showed a trend toward positive correlation with BCVA change (r = 0.195, 95% CI [−0.022, 0.394], *p* = 0.078, p_adj = 0.091), which did not survive FDR correction.

### 3.6. Multivariable Analysis of Predictors of BCVA Change

To assess whether baseline intraretinal HF burden was an independent predictor of BCVA change after controlling for confounders, multivariable linear regression was performed. The primary model (Model 2) included the baseline BCVA, intraretinal HF count, HRM width, EZD, HTC, and CMT in 67 eyes with complete covariate data. HRM height was excluded from the primary model because of its strong collinearity with HRM width. The overall model was statistically significant (F(6, 60) = 3.009, *p* = 0.012, adjusted R^2^ = 0.154). Two variables independently predicted BCVA change: baseline intraretinal HF count (β = −0.449, SE = 0.215, 95% CI [−0.879, −0.020], *p* = 0.041) and baseline HRM width (β = −0.003, SE = 0.001, 95% CI [−0.005, −0.001], *p* = 0.014). Baseline BCVA did not independently predict outcome (β = −0.079, *p* = 0.372), confirming that the HF–BCVA association was not driven by baseline severity. Full regression results are presented in Table 4.

Two sensitivity analyses were also conducted. In Model 1, including both HRM width and HRM height simultaneously (*n* = 65), HRM width remained the sole independent predictor (β = −0.003, *p* = 0.050), while HF burden showed a consistent negative trend without reaching significance (β = −0.379, *p* = 0.182). In Model 3, with both HRM dimensions removed (*n* = 84), the overall model was no longer significant (F(5, 78) = 1.075, *p* = 0.381, adjusted R^2^ = 0.005), and no individual predictor reached significance, confirming that HRM dimensions carry substantial prognostic information that, when omitted, is not recoverable from the remaining covariates in this cohort. Taken together, these analyses support HF burden as an independent predictor of BCVA change in the most parsimonious adequately powered model and demonstrate the robustness of HRM width as a structural predictor across all specifications.

### 3.7. Association Between HF and Hypertransmission into Choroid

The co-occurrence of elevated HF burden and HTC—both reflecting outer retinal and RPE disruption—was particularly prominent in Pattern 4, in which 64% of eyes had HTC at baseline, compared with 14–20% across Patterns 1–3 (Fisher exact *p* < 0.001, OR = 8.17). The predominance of Pattern 4 in our cohort (39.3%) and its strong association with poor visual outcomes is consistent with the real-world long-term data reported by Pauleikhoff et al., who demonstrated that Pattern 4 was associated with the development of complete RPE and outer retina atrophy and significantly reduced visual acuity at all timepoints compared with Patterns 1–3 [8]. Eyes with HTC at baseline had significantly higher intraretinal HF counts compared with eyes without HTC (*p* = 0.045).

### 3.8. Association Between EZD, HRM Dimensions and Visual Outcomes

No significant direct association between baseline EZD and BCVA change was observed (mean +1.4 letters in EZD-present eyes vs. +5.2 letters in EZD-absent eyes; *p* = 0.090), consistent with the AVENUE trial analysis, which framed EZD as a marker of structural severity rather than as an independent functional predictor. Baseline HRM width showed a strong negative correlation with BCVA change at M6 (Spearman r = −0.400, 95% CI [−0.584, −0.177], *p* = 0.001, p_adj = 0.003), and HRM height also correlated negatively with BCVA change (r = −0.392, 95% CI [−0.581, −0.164], *p* = 0.001, p_adj = 0.003). In the primary multivariable model, HRM width emerged as an independent predictor of BCVA change alongside intraretinal HF burden (β = −0.003, *p* = 0.014), confirming its role as a primary structural prognostic marker in this cohort.

## 4. Discussion

In this retrospective observational study of 84 treatment-naive nAMD eyes receiving bevacizumab via a treat-and-extend protocol, we demonstrate that intraretinal hyperreflective foci burden at baseline is a graded marker of HRM pattern severity and an independent predictor of visual outcomes at six months. In the primary multivariable model, both baseline intraretinal HF count and HRM width emerged as independent predictors of BCVA change, capturing complementary dimensions of outer retinal structural disruption.

### 4.1. HF as a Graded Marker of HRM Pattern Severity

The stepwise increase in the intraretinal HF counts across HRM Evolution Patterns 1 through 4 (median 0, 6, 4, and 8, respectively; *p* < 0.001) is a central finding of this study. This gradient suggests that HF burden reflects the degree of underlying outer retinal and RPE disruption that ultimately determines whether HRM undergoes complete boundary remodeling or persists with structural disorganization. Pattern 4 eyes—those with the worst outcomes and highest rates of intralesional HTC—also carried the greatest HF loads, consistent with a model in which multiple concurrent markers of RPE damage accumulate in the eyes least capable of effective structural remodeling.

Importantly, the signal was specific to intraretinal HF rather than subretinal HF (*p* = 0.192 across patterns at baseline; p_adj = 0.192). This compartmental specificity is biologically plausible: intraretinal HF, located within the photoreceptor and outer nuclear layers, are more likely to reflect microglial activation and RPE cell migration in direct proximity to the neovascular lesion, whereas subretinal HF may represent a more heterogeneous population of debris and cellular elements whose distribution is less tightly linked to RPE monolayer integrity.

A particularly notable observation was the transient peak in intraretinal HF in Pattern 3 eyes at M3 (median 12), which exceeded all other groups at that timepoint before resolving substantially by M6 (median 4), and which was statistically confirmed by Wilcoxon signed-rank testing (M0 → M3: *p* = 0.004; M3 → M6: *p* = 0.001). The biological basis of this transient increase remains to be established. One hypothesis is that it reflects a period of active cellular remodeling—including RPE cell migration and boundary consolidation—that precedes structural reorganization. However, direct validation of this mechanism would require supporting data, such as OCTA-based neovascular characterization, histologic correlation, or serial structural co-variables, which were not available in the present retrospective dataset. This observation should therefore be interpreted as a hypothesis-generating finding warranting prospective investigation.

### 4.2. HF Burden and Visual Outcomes

The negative correlation between the baseline intraretinal HF count and BCVA change at M6 (r = −0.300, 95% CI [−0.483, −0.092], p_adj = 0.010) extends the prior literature linking HF to visual prognosis in nAMD [13,14]. Eyes with high HF burden (>5 intraretinal foci) gained on average only 0.8 ETDRS letters over six months, compared with 4.8 letters in low-burden eyes (*p* = 0.008). While the absolute differences are modest, they reflect a clinically meaningful divergence in treatment response at the population level and are consistent with the Pattern 4 outcomes described by Yu et al. in the AVENUE trial [7].

In the primary multivariable regression model (*n* = 67), baseline intraretinal HF count emerged as an independent predictor of BCVA change (β = −0.449, 95% CI [−0.879, −0.020], *p* = 0.041), alongside HRM width (β = −0.003, *p* = 0.014). In a sensitivity analysis including both HRM dimensions (Model 1, *n* = 65), HF burden showed a consistent negative direction (β = −0.379) that did not reach statistical significance (*p* = 0.182), reflecting collinearity between HRM width and height and reduced power with seven simultaneous covariates. The absence of confounding by baseline BCVA across all model specifications (*p* = 0.372 to 0.689) confirms that the HF–BCVA association was not driven by differential baseline severity. Taken together, these findings support HF count and HRM width as complementary, independently contributing markers of outer retinal disruption, each capturing a different dimension of disease burden [16].

The additional finding that HRM width at baseline was more strongly correlated with BCVA change than HF count (r = −0.400, 95% CI [−0.584, −0.177], p_adj = 0.003) is consistent with prior reports that the horizontal extent of HRM—reflecting the area of the RPE and photoreceptor involvement—is a stronger functional predictor than lesion height [7,10].

### 4.3. HF Dynamics as a Longitudinal Biomarker

A trend toward correlation between longitudinal intraretinal HF reduction and visual improvement was observed (r = 0.195, 95% CI [−0.022, 0.394], *p* = 0.078, p_adj = 0.091), which did not survive FDR correction in the present cohort. The minimum detectable correlation at 80% power for *n* = 84 is r = 0.302, indicating that the observed effect size (r = 0.195) falls below the detectable threshold for this sample size. The direction and magnitude of the association nevertheless suggest that serial HF quantification may provide a structural signal of treatment response, and prospective validation in larger cohorts with automated volumetric HF quantification is warranted.

This concept aligns with the emerging view of HF as dynamic, treatment-responsive biomarkers rather than static baseline risk factors [11]. If validated prospectively, serial HF monitoring could serve as an intermediate OCT endpoint in nAMD trials, bridging the gap between structural imaging findings and functional outcomes.

### 4.4. Ellipsoid Zone Disruption and Pattern Severity

The EZD frequencies increased progressively across HRM patterns (24%, 67%, 67%, and 85% for Patterns 1–4, respectively; *p* < 0.001), consistent with the findings of Yu et al., who similarly reported higher EZD rates in more severe HRM evolution groups [7,8]. No significant direct association between baseline EZD and BCVA change was observed in our cohort (*p* = 0.090), consistent with the AVENUE trial analysis, which framed EZD as a marker of structural severity rather than as an independent functional predictor. The combination of elevated intraretinal HF burden and EZD at baseline may identify eyes with both active outer retinal inflammation and established photoreceptor structural damage—a potentially high-risk phenotype for incomplete HRM-BR and poor visual recovery. Prospective studies incorporating multivariate modeling of the HF, EZD, HTC, and HRM dimensions are needed to determine the independent and combined contributions of these biomarkers to functional prognosis in nAMD.

### 4.5. HF and Hypertransmission into Choroid

The co-occurrence of elevated intraretinal HF with HTC (*p* = 0.045) is a further finding of interest. HTC is an established proxy for RPE atrophy and has previously been shown to predict incomplete HRM-BR and worse BCVA in nAMD [7]. In the AVENUE trial, intralesional HTC at month 9 was present in 69.2% of Pattern 4 eyes versus 20.8% of Pattern 3 eyes—a distribution closely mirrored in our cohort, where HTC was present in 64% of Pattern 4 eyes at baseline, compared with 20% in Pattern 3. The current findings suggest that intraretinal HF and HTC may share a common pathological substrate—specifically, disruption of the RPE monolayer—and that their co-occurrence may identify a subgroup of eyes at particularly high risk of poor structural and functional outcomes. Nanoparticle-based and exosome-mediated drug delivery systems targeting RPE dysfunction represent an emerging translational direction in this context, and future studies examining HF and HTC as stratification markers in such trials may be of interest [16].

## 5. Conclusions

Intraretinal hyperreflective foci burden at baseline is a graded marker of HRM pattern severity in nAMD and an independent predictor of worse visual outcomes following anti-VEGF treatment with bevacizumab. In the primary multivariable analysis, both baseline intraretinal HF count (β = −0.449, *p* = 0.041) and HRM width (β = −0.003, *p* = 0.014) independently predicted BCVA change, demonstrating that the HF and HRM dimensions capture complementary dimensions of outer retinal structural disruption. A statistically confirmed transient peak in intraretinal HF at month 3 in Pattern 3 eyes (*p* = 0.004 for M0 → M3; *p* = 0.001 for M3 → M6) represents a novel observation that warrants mechanistic investigation. The EZD frequency increased progressively with the HRM pattern severity, consistent with the prior literature, though no direct EZD-BCVA association was demonstrated. The co-occurrence of elevated HF burden with hypertransmission into choroid supports a model of converging RPE disruption markers in the most severe HRM evolution subtype. Larger prospective studies incorporating volumetric HF quantification, OCTA-based MNV subtyping, and longer follow-up are warranted to validate and extend these findings.

## 6. Limitations

This study has several limitations that should be considered when interpreting the findings. First, the retrospective single-center design introduces potential selection bias, as patients requiring complete imaging at all three timepoints may represent a more compliant or clinically stable subgroup, and the findings may not be fully generalizable to broader clinical populations. The relatively high proportion of Pattern 4 eyes (39.3%) in this cohort may reflect referral or practice patterns specific to the study setting and may differ from the distribution observed in randomized trial cohorts.

Second, HF quantification was restricted to five fovea-centered B-scans per timepoint using observer-assisted counting in ImageJ, despite the availability of full 3D macular volume data. This approach may underestimate the total HF burden and introduces spatial sampling bias, particularly in eyes with heterogeneous or eccentric lesions. Automated volumetric HF quantification across the full scan would provide more spatially comprehensive and reproducible measurements and should be prioritized in future studies. Although inter-rater agreement was good to excellent (ICC ≥ 0.88 for intraretinal HF), the semi-manual nature of the counting process limits scalability.

Third, all patients received bevacizumab under a treat-and-extend protocol; no bevacizumab-naive control arm or comparison with other anti-VEGF agents (aflibercept, ranibizumab, brolucizumab) was available. OCTA-based macular neovascularization (MNV) subtype classification was not performed, precluding stratification by lesion type (type 1, 2, or 3 MNV), which is a known modulator of HRM evolution and visual outcomes. The Revo FC spectral-domain OCT device used in this study has not been validated against other commercially available OCT platforms for HF quantification; generalizability of the specific HF count thresholds reported here to studies using other devices should be assessed cautiously.

Fourth, the six-month follow-up period is relatively short for nAMD, precluding assessment of longer-term structural outcomes, including macular atrophy progression, subretinal fibrosis development, and long-term visual stability. Key structural endpoints such as complete RPE and outer retina atrophy—which are among the most clinically meaningful outcomes of nAMD—were not captured within this timeframe. Fifth, multivariable regression was performed in 65–84 eyes depending on covariate availability, with the primary model including 67 eyes. The model explains approximately 15% of the variance in BCVA change (adjusted R^2^ = 0.154 in the primary model), indicating that important predictors beyond those assessed—including MNV subtype, treatment adherence, and genetic factors—remain unaccounted for, and larger datasets are needed to resolve subtle independent contributions of correlated structural biomarkers.

## Figures and Tables

**Table 1:** Baseline and outcome characteristics by HRM evolution pattern (*n* = 84).

Variable	Pattern 1 (*n* = 21)	Pattern 2 (*n* = 15)	Pattern 3 (*n* = 15)	Pattern 4 (*n* = 33)
BCVA baseline, ETDRS letters, mean ± SD	62.0 ± 20.7	54.7 ± 12.8	36.0 ± 13.7	30.9 ± 14.2
BCVA at M6, ETDRS letters, mean ± SD	65.9 ± 22.6	66.9 ± 9.0	42.0 ± 16.9	27.3 ± 14.7
**BCVA change M0–M6, mean ± SD**	+3.9 ± 7.1	+12.2 ± 11.4	+6.0 ± 8.9	**−3.6 ± 6.2**
HTC present at baseline, n (%)	3 (14%)	3 (20%)	3 (20%)	**21 (64%)**
ELM disruption present at baseline, n (%)	7 (33%)	10 (67%)	11 (73%)	26 (79%)
EZD present at baseline, n (%)	5 (24%)	10 (67%)	10 (67%)	28 (85%)

BCVA—best-corrected visual acuity; ELM disruption—disruption of external limiting membrane; EZD—ellipsoid zone disruption; HTC—hypertransmission into choroid; SD—standard deviation. Bold values indicate highest-risk pattern (Pattern 4). Kruskal–Wallis test was used for continuous variables and Fisher exact test for categorical variables across four HRM patterns.

**Table 2:** Hyperreflective foci counts by compartment and HRM evolution patterns across timepoints (*n* = 84).

HF Variable	Pattern 1 (*n* = 21)	Pattern 2 (*n* = 15)	Pattern 3 (*n* = 15)	Pattern 4 (*n* = 33)	*p* *	p_adj †
Intraretinal HF M0, median (IQR)	0 (0–2)	6 (4–6)	4 (2.5–9)	8 (2–10)	**<0.001**	**<0.001**
Intraretinal HF M3, median (IQR)	0 (0–1)	3 (1–3)	12 (6–16)	4 (2–10)	**<0.001**	**<0.001**
Intraretinal HF M6, median (IQR)	0 (0–0)	1 (0–3)	4 (3–7)	3 (0–10)	**<0.001**	**<0.001**
HF subretinal M0, median (IQR)	1 (0–3)	1 (1–7)	2 (1–9)	4 (0–7)	0.192	0.192
HF subretinal M3, median (IQR)	0 (0–0)	1 (0–2)	5 (3–9)	1 (0–5)	**<0.001**	**<0.001**
HF subretinal M6, median (IQR)	0 (0–1)	1 (0–2)	2 (0–4)	1 (0–4)	**0.038**	0.053
Intraretinal HF change M0 → M6, mean ± SD	−2.2 ± 4.3	−2.0 ± 3.3	+0.3 ± 6.6	−3.6 ± 6.6	—	—

* Kruskal–Wallis test across all four patterns. † FDR-adjusted *p*-value (Benjamini–Hochberg). Post hoc Dunn–Bonferroni pairwise comparisons for intraretinal HF M0: P1 vs. P2, *p* = 0.0004; P1 vs. P3, *p* = 0.0005; P1 vs. P4, *p* = 0.0001; P2 vs. P4, *p* = 0.043; P3 vs. P4, *p* = 0.372 (NS). Wilcoxon signed-rank within-group comparisons for Pattern 3: M0 → M3, *p* = 0.004; M3 → M6, *p* = 0.001; M0 → M6, *p* = 0.826. Bold values indicate statistical significance (*p* < 0.05). HF—hyperreflective foci; IQR—interquartile range; NS—not significant; SD—standard deviation.

**Table 3:** Spearman rank correlations between HF burden, HRM dimensions, and BCVA outcomes, with 95% confidence intervals and FDR-adjusted *p*-values.

Association	Spearman r	95% CI	*p* Value	p_adj †
HRM pattern vs. intraretinal HF M0	**0.471**	[0.286, 0.623]	**<0.001**	**<0.001**
Intraretinal HF M0 vs. BCVA change	**−0.300**	[−0.483, −0.092]	**0.006**	**0.010**
Subretinal HF M0 vs. BCVA change	−0.177	[−0.377, 0.039]	0.107	0.107
Total HF burden M0 vs. BCVA change	**−0.248**	[−0.439, −0.036]	**0.023**	**0.032**
Intraretinal HF change M0 → M6 vs. BCVA change	0.195	[−0.022, 0.394]	0.078	0.091
HRM width at baseline vs. BCVA change	**−0.400**	[−0.584, −0.177]	**0.001**	**0.003**
HRM height at baseline vs. BCVA change	**−0.392**	[−0.581, −0.164]	**0.001**	**0.003**

† FDR-adjusted *p*-value (Benjamini–Hochberg method). Bold values indicate p_adj < 0.05; 95% CIs derived by Fisher z-transformation. Bold values indicate statistical significance (*p* < 0.05). BCVA—best-corrected visual acuity; HF—hyperreflective foci; HRM—hyperreflective material.

**Table 4:** Multivariable linear regression: predictors of BCVA change from baseline to month 6—primary model (Model 2) with sensitivity analyses.

Variable	Model 1 (*n* = 65): All Covariates	Model 2 (*n* = 67): Primary HRM Width Only	Model 3 (*n* = 84): No HRM Dimensions
Baseline BCVA	−0.050 [−0.242, 0.141] *p* = 0.600	−0.079 [−0.254, 0.096] *p* = 0.372	+0.029 [−0.116, 0.174] *p* = 0.689
Intraretinal HF at M0	−0.379 [−0.940, 0.183] *p* = 0.182	**−0.449 [−0.879, −0.020] *p* = 0.041**	−0.252 [−0.664, 0.160] *p* = 0.226
HRM width at M0	**−0.003 [−0.005, −0.000] *p* = 0.050**	**−0.003 [−0.005, −0.001] *p* = 0.014**	—
HRM height at M0	−0.009 [−0.042, 0.023] *p* = 0.576	—	—
EZD (present vs. absent)	−5.229 [−11.140, 0.682] *p* = 0.082	−4.926 [−10.500, 0.649] *p* = 0.082	−2.108 [−7.291, 3.075] *p* = 0.421
HTC (present vs. absent)	−2.070 [−7.467, 3.327] *p* = 0.446	−1.635 [−6.719, 3.450] *p* = 0.523	−0.921 [−5.978, 4.136] *p* = 0.718
CMT at M0	+0.004 [−0.020, 0.027] *p* = 0.752	−0.001 [−0.020, 0.018] *p* = 0.925	+0.000 [−0.014, 0.015] *p* = 0.984
Model fit	F(7, 57) = 2.626 *p* = 0.020, adj R^2^ = 0.151	F(6, 60) = 3.009 *p* = 0.012, adj R^2^ = 0.154	F(5, 78) = 1.075 *p* = 0.381, adj R^2^ = 0.005

Dependent variable: BCVA change M0→M6 (ETDRS letters). Model 1 (*n* = 65): all 7 covariates including both HRM dimensions; F(7, 57) = 2.626, *p* = 0.020, adjusted R^2^ = 0.151. Model 2 (primary, *n* = 67): HRM width only; F(6, 60) = 3.009, *p* = 0.012, adjusted R^2^ = 0.154. Model 3 (*n* = 84): no HRM dimensions; F(5, 78) = 1.075, *p* = 0.381, adjusted R^2^ = 0.005. Bold values indicate *p* < 0.05. β—unstandardized beta coefficient; SE—standard error; CI—confidence interval. Bold values indicate *p* < 0.05. BCVA—best-corrected visual acuity; CMT—central macular thickness; EZD—ellipsoid zone disruption; HF—hyperreflective foci; HRM—hyperreflective material; HTC—hypertransmission into choroid.

## Data Availability

Data are available from the corresponding author upon reasonable request.

## References

[1] Wong W.L., Su X., Li X., Cheung C.M.G., Klein R., Cheng C.-Y., Wong T.Y. (2014). Global prevalence of age-related macular degeneration and disease burden projection for 2020 and 2040: A systematic review and meta-analysis. Lancet Glob. Health.

[2] Teper S., Ledwoń D., Romaniszyn-Kania P., Sendecki A., Tuszy A., Nycz J., Mitas A.W., Figurska M., Wyłęgała E., Rękas M. (2025). Twelve-month outcomes of anti-VEGF therapy for nAMD with brolucizumab, aflibercept, and ranibizumab in the Polish National Registry: A multicenter database study. J. Clin. Med..

[3] Nawash B., Ong J., Driban M., Hwang J., Chen J., Selvam A., Mohan S., Chhablani J. (2023). Prognostic optical coherence tomography biomarkers in neovascular age-related macular degeneration. J. Clin. Med..

[4] DeCroos F.C., Toth C.A., Stinnett S.S., Heydary C.S., Burns R., Jaffe G.J. (2012). Optical coherence tomography grading reproducibility during the Comparison of Age-Related Macular Degeneration Treatments Trials. Ophthalmology.

[5] Shah V.P., Shah S.A., Mrejen S., Freund K.B. (2014). Subretinal hyperreflective exudation associated with neovascular age-related macular degeneration. Retina.

[6] Ying G., Kim B.J., Maguire M.G., Huang J., Daniel E., Jaffe G.J., Grunwald J.E., Blinder K.J., Flaxel C.J., Rahhal F. (2014). Sustained visual acuity loss in the Comparison of Age-Related Macular Degeneration Treatments Trials. JAMA Ophthalmol..

[7] Yu S., Bachmeier I., Hernandez-Sanchez J., Garcia Armendariz B., Ebneter A., Pauleikhoff D., Chakravarthy U., Fauser S. (2023). Hyperreflective material boundary remodeling in neovascular age-related macular degeneration. Ophthalmol. Retina.

[8] Pauleikhoff D., Yu S., Bachmeier I., Armendariz B.G., Bormann E., Pauleikhoff L. (2025). Hyperreflective material evolution patterns during long-term anti-VEGF therapy in neovascular age-related macular degeneration. Graefe’s Arch. Clin. Exp. Ophthalmol..

[9] Frizziero L., Midena G., Danieli L., Torresin T., Perfetto A., Parrozzani R., Pilotto E., Midena E. (2025). Hyperreflective retinal foci (HRF): Definition and role of an invaluable OCT sign. J. Clin. Med..

[10] Mat Nor M.N., Green C.R., Squirrell D., Acosta M.L. (2025). Retinal hyperreflective foci are biomarkers of ocular disease: A scoping review with evidence from humans and insights from animal models. J. Ophthalmol..

[11] Abri Aghdam K., Pielen A., Framme C., Junker B. (2015). Correlation between hyperreflective foci and clinical outcomes in neovascular age-related macular degeneration after switching to aflibercept. Investig. Ophthalmol. Vis. Sci..

[12] Verma N., Khare D., Poe A.J., Amador C., Ghiam S., Fealy A., Ebrahimi M., Shieh D., Bedrat A., Rhee H. (2023). MicroRNA and protein cargos of human limbal epithelial cell-derived exosomes and their regulatory roles in limbal stromal cells of diabetic and non-diabetic corneas. Cells.

[13] Hsia Y., Yang C.-H., Hsieh Y.-T., Yang C.-M., Ho T.-C., Lai T.-T. (2020). Hyperreflective foci in predicting the treatment outcome of antivascular endothelial growth factor in neovascular age-related macular degeneration. Graefe’s Arch. Clin. Exp. Ophthalmol..

[14] Parolini F., Pilotto E., Midena E., Midena G. (2026). Inflammatory hyperreflective retinal foci: An OCT biomarker of neuroinflammation in geographic atrophy. J. Clin. Med..

[15] Sadda S.R., Guymer R., Holz F.G., Schmitz-Valckenberg S., Curcio C.A., Bird A.C., Blodi B.A., Bottoni F., Chakravarthy U., Chew E.Y. (2018). Consensus definition for atrophy associated with age-related macular degeneration on OCT: Classification of atrophy report 3. Ophthalmology.

[16] Verma N., Arora S., Singh A.K., Ahmed J. (2025). Unlocking the potential of exosomes ‘extracellular vesicles’: Drug delivery advancements and therapeutics in ocular diseases. RSC Pharm..

